# Road Traffic Anomaly Detection via Collaborative Path Inference from GPS Snippets

**DOI:** 10.3390/s17030550

**Published:** 2017-03-09

**Authors:** Hongtao Wang, Hui Wen, Feng Yi, Hongsong Zhu, Limin Sun

**Affiliations:** 1 Beijing Key Laboratory of IOT Information Security, Institute of Information Engineering, CAS, Beijing 100093, China; wanghongtao@iie.ac.cn (H.W.); wenhui@iie.ac.cn (H.W.); yifeng@iie.ac.cn (F.Y.); sunlimin@iie.ac.cn (L.S.); 2 School of Cyber Security, University of Chinese Academy of Sciences, Beijing 100049, China

**Keywords:** road anomaly detection, path inference, tensor decomposition

## Abstract

Road traffic anomaly denotes a road segment that is anomalous in terms of traffic flow of vehicles. Detecting road traffic anomalies from GPS (Global Position System) snippets data is becoming critical in urban computing since they often suggest underlying events. However, the noisy and sparse nature of GPS snippets data have ushered multiple problems, which have prompted the detection of road traffic anomalies to be very challenging. To address these issues, we propose a two-stage solution which consists of two components: a *Collaborative Path Inference (CPI)* model and a *Road Anomaly Test (RAT)* model. *CPI* model performs path inference incorporating both static and dynamic features into a *Conditional Random Field (CRF)*. Dynamic context features are learned collaboratively from large GPS snippets via a tensor decomposition technique. Then *RAT* calculates the anomalous degree for each road segment from the inferred fine-grained trajectories in given time intervals. We evaluated our method using a large scale real world dataset, which includes one-month GPS location data from more than eight thousand taxicabs in Beijing. The evaluation results show the advantages of our method beyond other baseline techniques.

## 1. Introduction

Smart vehicles equipped with communication modules and various types of sensors are becoming an important deployment scenario of IoT (Internet of Things) technology. Harvesting data collected by sensors mounted on vehicles has been quite fruitful in urban computing [1] and a lot of new applications have been proposed. Among them, road traffic anomaly detection is critical since they often suggest underlying events, e.g., accidents, traffic controls, terrorist attacks, protests, disasters, violent riots, to name a few. The effective detection of road traffic anomaly can not only provide knowledge for applications such as vehicular ad hoc networks [2,3] but, more importantly, facilitate the government to deal with the possible emergencies timely.

A road traffic anomaly behaves as a state for a road segment in a specific time when the volume of vehicles on the segment is significantly unusual. To effectively monitor and summarize the volume of vehicles on each road segment, traditional techniques are developed including video surveillance [4] and radio frequency identification devices (RFID) [5]. However, in the urban environment, usually only a small number of road segments have deployed video surveillance system or RFID readers, leaving the data of uncovered segments missing. With the profusion of GPS sensors mounted on vast amount of vehicles, the big location data generated from these sensors will provide a citywide solution on traffic flow monitoring and anomaly detection.

Location data collected by vehicles are often in the form of “GPS snippet” which is defined as a set of timestamped GPS coordinates [6]. Directly using GPS snippets data to find road traffic anomalies is a particularly challenging task for several reasons. First, the nature of GPS devices makes measured locations very noisy, where errors may range from meters to tens of meters. Thus the GPS measurements may not lie in road segments. Second, although the number of vehicles is large, they often report location records in low-sample-rate time intervals (e.g., in the order of 30–60 s). As a result, many GPS snippets are very sparse, namely, too coarse-grained for mining road traffic anomalies. To address these issues, this paper reconstructs fine-grained trajectories from coarse-grained GPS snippets via a path inference model and then find road traffic anomalies from the inferred trajectories.

**Figure 1:**
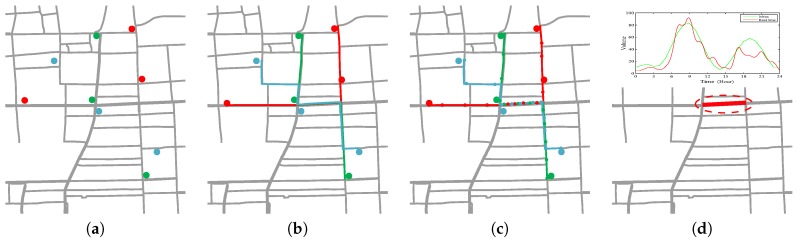
Road Anomaly Detection. (**a**) GPS Snippets; (**b**) Full Paths; (**c**) Trajectories; (**d**) Anomaly Detection.

For example, Figure 1 shows the basic process of road anomaly detection. In Figure 1a, there are three vehicles whose GPS snippets are drawn in red, blue and green respectively. Then the full paths (the solid lines of different colors in Figure 1b) of all vehicles are inferred. Next the fine-grained trajectories of vehicles are reconstructed. In Figure 1c, the smaller dots on each path of vehicles are the possible location at given time. According to the fine-grained trajectories, we count the volume of vehicles on each road segment and detect the suspicious segments through volume data analytics (Figure 1d).

Reconstructing fine-grained trajectories from noisy and sparse GPS snippets, is often referred to as *map matching* [7] or *path inference* [8]. However, previous studies often overlook road dynamic context information which is particularly important for path inference. Combined with dynamic context features (e.g., popularity and average velocity of road segments), path inference algorithms could evaluate the latent path selections more preciously. Recent advances in data mining domain, especially the collaborative filter techniques [9], have provided methods of inferring latent dynamic context from snippets data. The basic idea of collaborative filter is to measure the temporal and spatial “similarities” between vehicles to infer the probability a vehicle appearing in one specific road segment at a given time span. In other words, it can discover latent factors of both road segments and vehicles, that together determine the generation of GPS snippets in different time spans.

In this paper, we propose a method for road traffic anomaly detection via collaborative path inference from GPS snippets. Our method consists of two main components: a *Collaborative Path Inference (CPI)* model and a *Road Anomaly Detection (RAD)* algorithm. Our *CPI* model not only utilize static features for path inference, but learning dynamic features collaboratively via a tensor decomposition technique. We summarize the contributions of our work as follows:

- *Collaborative path inference.* *CPI* model performs collaborative path inference incorporating both static and dynamic feature into a *Conditional Random Field (CRF)*, and then reconstructs sparse GPS snippets to fine-grained trajectories;
- *Dynamic feature learning.* We aim to collaboratively learn dynamic context features hidden in data via tensor decomposition [10] technique. To tackle the data sparsity problem in the GPS snippets dataset, we exploit two normalization terms, which are robust and can avert over-fitting;
- *Road anomaly detection.* We calculate the anomalous degree for each road segment from counting the volumes of fine-grained trajectories in given time intervals. Two kinds of road anomalies are defined in this paper and we devise an algorithm to detect them effectively;
- *Real evaluation.* We evaluate our solution using a real world dataset including a large number of taxi traces. Experimental results show that our solutions are effective in path inference and road anomaly detection especially under low sampling rate between GPS locations.

The rest of the paper is organized as follows. We briefly review related work in Section 2. In Section 3, we introduce some concept in this paper and give an overview of our method. Section 4 defines the path inference problem and presents our CRF model. We also propose a collaborative dynamic feature learning method under tensor decomposition in this section. In Section 5, we recognize two types of road anomalies and perform a statistic test on the volume of road segments for detection. In Section 6, we evaluate our solution using a large-scale dataset and report the analysis results. Finally in Section 7 we conclude the paper and envisage the future work.

## 2. Related Work

### 2.1. Path Inference

With the growing popularity of GPS-based applications such as VANETs [3,11], the requirements for fine-grained trajectories are more ubiquitous. The problem of mapping GPS points onto a map is first studied in [12], which uses several simple approaches to match GPS points to nearest road segments. We categorize existing methods into two class: deterministic and probabilistic. Deterministic approaches associate each observation to a segment in road network. They utilize geometric information of road network by considering the shape of the roads [13], or the connectivity and contiguity information [7]. In [14], frèchet distance is used to match partial trajectories to road segments. All those algorithms are very fast, however, they are sensitive to noisy GPS observations.

To overcome the uncertainty of observations, many probabilistic algorithms have been proposed by adopting the idea of particle filter [15], Kalman filter [16] and Interactive-Voting [17]. Under the assumption of *Markov* independence relations, Hidden Markov Model (HMM) [18] and Conditional Random Fields (CRF) [8] have been explored. Both HMM and CRF need to utilize various features for designing the transition probability of states, which encourage the weight learning algorithm using inverse reinforcement learning [19]. However, these algorithms show that the performance is poor when the intervals of GPS observations exceed 5 min [18].

One of the problems of HMM and CRF is that they use context-unaware features when computing the transition probability. These features could not reflect the real traffic condition spatially and temporally. In this paper, we introduce the collaborative method [9] to extract dynamic context-aware features from observations. Two influential collaborative filter techniques are matrix factorization and tensor decomposition [10], which have become increasingly popular recently. Tensor decomposition is adopted to process mobile network data for a number of data mining tasks, such as travel time estimation [20], demographic attributes inference [21], social networks [22], and link pattern prediction [23], to name a few. In this paper, we utilize incomplete GPS snippets with different time intervals to extract dynamic features using tensor decomposition, and then construct a CRF model for path inference.

### 2.2. Road Anomaly Detection

Over the past decades, road traffic patterns or anomaly detections have been extensively studied [24,25]. Many traditional tools are developed such as video surveillance [4], sensor networks [26] and RFID [5] technique. With the profusion of GPS devices driven location-based techniques, the spatio-temporal data have ushered road anomaly detection problems very challenging and attracted many researches. Given GPS observations, one class of anomaly detection technique is to discover individual suspicious vehicles such as wandering round vehicles [27]. The other class is road traffic based anomaly detection, whose aim is to monitor the inflows or outflows of road segments and extrapolate possible events from volumes of stay vehicles. For example, the authors of [28] used GPS data from taxicabs to find out the emergence of unexpected or abnormal traffic behavior. The authors of [29] focused on inferring the root cause in road traffic anomalies, and the authors of [30] discovered the spatio-temporal causal interactions from traffic data streams. However, all these studies assume the GPS data are fine-grained. In this paper, we detect road traffic anomalies from original GPS snippets by inferring fine-grained trajectories.

To judge the anomaly degree, threshold-based methods always set the upper and lower limits learned from historical data [31,32]. An alternative way to determine the anomaly degree is Likelihood Ratio Test (LRT) [33] which is a statistical hypothesis test based method. One of the advantages of LRT technique is that it doesn’t need a hard threshold, but a statistical significance level. Thus we adopt LRT to detect two kinds of road traffic anomalies defined in this paper.

## 3. Background

In this section, we define several concepts for path inference and anomaly detection used throughout this paper and give problem definitions of road traffic anomaly detection. Finally we overview the system framework of our method.

### 3.1. Path Inference

**Figure 2:**
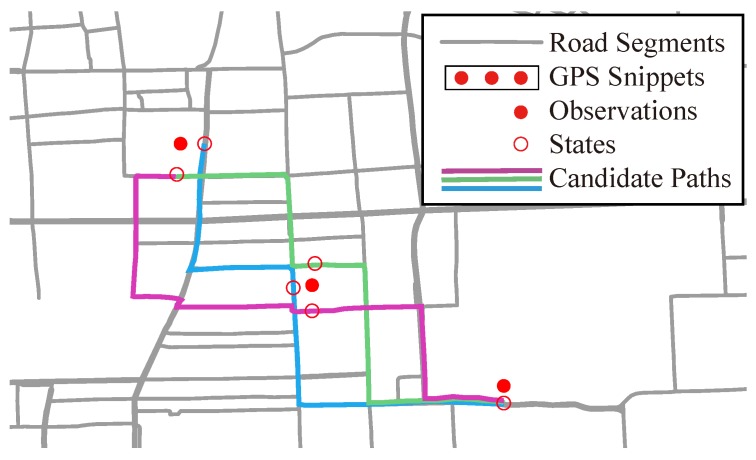
Concepts on path inference.

We first introduce related concepts in this subsection. Figure 2 shows an example to illustrate these concepts.

**Road Network.** A *Road Network* is an undirected graph N={V,E}, where E is a set of edges denoting the road segments and V is a set of vertices representing the intersections or terminal points of the road segments. Each road segment e∈E is associated with a number of attributes such as segment ID, start node, end node, and length.

**GPS Observation and Snippet.** Millions of GPS measurements can be organized by vehicles within a time span [1,T]. A GPS observation *g* is represented as a triple: *(latitude, longitude, time stamp)*. For a given vehicle, we denote a GPS snippet with *n* observations as $G=\{g^{1},g^{2},\cdots ,g^{n}\}$ where $g^{t}$ denotes the observation at *t*. The time span between two neighbouring observations is sampling interval, which can be different for different vehicles.

**State.** Due to the noisy nature of GPS measurements, state x=(l,o) is a projection of observation *g* to a road segment *l*, where *o* is the offset of projection from the start node of *l*. As one observation $g^{t}$ can be projected to a number of roads, we can get $I^{t}$ different candidate states $x^{t}=\left\{x_{1}^{t},x_{2}^{t},\cdots ,x_{I^{t}}^{t}\right\}$.

**Path.** Between two adjacent states $x_{i}^{t}\in x^{t}$ and $x_{i^{\prime }}^{t+1}\in x^{t+1}$, only a small number of road segments can be taken by a vehicle. We denote the set of candidate path selections between two consecutive observations $g^{t}$ and $g^{t+1}$ as $\Omega^{t}=\left\{\xi_{1}^{t},\xi_{2}^{t},\cdots ,\xi_{J^{t}}^{t}\right\}$, where $J^{t}$ is the number of candidate selections at time stamp *t*. Note that a candidate path selection $\xi_{j}^{t}$ may be one road segment or consists of several conjoint road segments. If the distance of two observations is close (e.g., on one segment), $\xi_{j}^{t}$ is a road segment. Otherwise if this distance is very far, then $\xi_{j}^{t}$ can be a set of conjoint road segments.

Then we define *Path* for GPS snippet *G* as a sequence of latent states and road segments, starting and ending with a state, denoted as $\tau =x^{1}\xi^{1}x^{2}\xi^{2}\cdots \xi^{n-1}x^{n}$, where $x^{t}$ and $\xi^{t}$ are element of $x^{t}$ and $\Omega^{t}$ respectively. We denote the path space as T, whose dimensions are $I^{1}\times J^{1}\times I^{2}\times J^{2}\cdots J^{n-1}\times I^{n}$.

In Figure 2, three locations labeled in red solid circles constitute a GPS snippet, in which each location is defined as observation. Each observation may generate a small different number of states, which are labeled as red hollow circles. A path is a set of road segments passing through states for every observations, e.g., three candidate paths colored in purple, blue and green. The path inference problem then can be defined as:

**Definition 1** **(Path Inference).** *Given the road network N and the raw GPS snippets G,* Path Inference is to find the most likely path $\tau^{*}$ in the latent variable space T for the vehicle that generates GPS snippets G.

Given an inferred full path, our next aim is to reconstruct a fine-grained trajectory for every snippet. We consider the trajectory of a path to be a dense synthetic GPS snippet, which is formally defined as:

**Trajectory.** Given a time interval [1,T], for every granularity $1<t_{1}<t_{2}<\cdots <t_{m}<T$, a trajectory is a set of consecutive locations on road segments $L=\{l_{1},l_{2},\cdots ,l_{m}\}$, where $l_{i}=\left(t_{i},r_{i},o_{i}\right)$, $t_{i}$ is a specific time stamp, $r_{i}$ indicates a road segment in road network and $o_{i}$ is the offset from the start node of the segment.

Note that once the full paths of vehicles are inferred, the trajectories can be estimated from paths. We leave the details in Section 5.1.

### 3.2. Road Anomaly Detection

After the process of path inference, the fine-grained trajectories for all vehicles are reconstructed. We can naturally count the volumes staying on every segment e∈E of road network N. Specifically, segment *e* is associated with a set of vehicle volumes during recent time interval [1,T]. From all volume sets of road segments in E, we aim to detect two types of anomalies.

- **Self-Evolving Anomaly (SEA).** Given time interval [1,T], assume all road segments have the same type of expected volume distribution. A *Self-Evolving Anomaly* is a road segment e∈E, such that in time interval [1,T] the values of associated volume set deviate from the expected distribution according to its historical values.
- **Context-Evolving Anomaly (CEA).** A *Context-Evolving Anomaly* is a road segment e∈E, such that in time interval [1,T] the values of associated volume set deviate from the values of its connected neighbors in a region. Note that since we focus on road segments within local area, even two remote road segments could behave similarly but we leave this out of scope.

**Figure 3:**
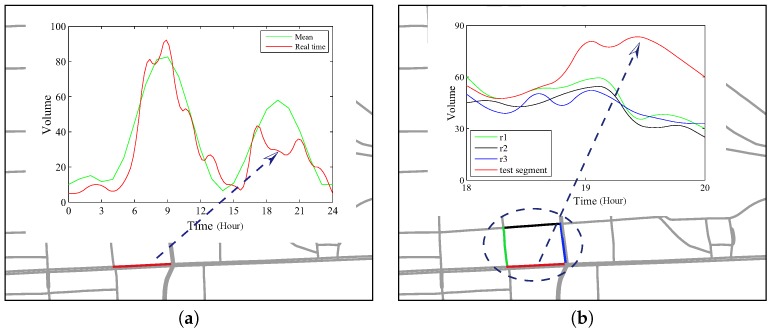
Two types of road anomalies. (**a**) Self-Evolving Anomaly; (**b**) Context-Evolving Anomaly.

Figure 3 shows an example for the two types of anomalies. In Figure 3a, the segment in red is a SEA in time interval [18,20]. We can see the volumes of staying vehicles are evidently lower than the mean values. However, in Figure 3b, the segment in red color is not a SEA, but a CEA in the context of the selected region. The volumes of the test segment in time interval [19,20] are much higher than other segments in the region. Both SEA and CEA anomalies may suggest an event such as a parade or traffic control. Therefore, the goal of this paper is to find out road traffic anomalies of both types. We define this problem as follows.

**Definition 2** **(Road Traffic Anomaly Detection).** *Given a road network N and large amount of GPS snippets in recent time interval [1,T], detect all the Self-Evolving Anomalies and Context-Evolving Anomalies from segments set E.*

### 3.3. System Framework

**Figure 4:**
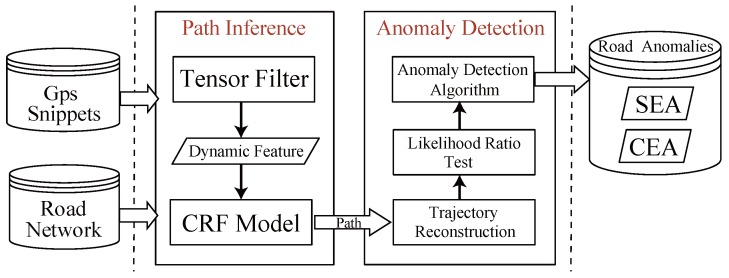
Overview of our method.

Figure 4 shows the framework of our method. Our method consists of two main components: a *Path Inference* component and an *Anomaly Detection* component.

The first component, namely path inference, is to complete full paths from incomplete or sparse GPS snippets, which are the inputs of our model. We separate the path inference process into two phases. In the first phase, road network and raw GPS snippets are filled into a three order tensor which is also incomplete. A tensor decomposition is employed to construct a completed tensor and extract collaborative dynamic features. To deal with data sparseness, reasonable regularization terms are added to perform the tensor decomposition procedure. In the second phase, dynamic features, as well as the static features, are filled into a conditional random field (CRF) model to run the path inference algorithm. The outputs of path inference process are full paths for all snippets of vehicles.

The goal of second component is to detect road traffic anomalies from the completed full paths of vehicles. First, trajectory reconstruction transforms paths to fine-grained trajectories such that the volumes of staying vehicles on all segments at any specific time are derived. Then second, given recent volume sets of road segments, a Likelihood Ratio Test (LRT) method can determine whether a segment is a road traffic anomaly. Finally, the *RN-Scan* algorithm searches the whole road network to find out all possible anomalies including *SEA* and *CEA*. We show the detailed contents of our method in the following sections.

## 4. Path Inference

In this section, we firstly propose a probabilistic graphical model for path inference. Then we illustrate how to learn dynamic features using tensor decomposition technique. Under both static and dynamic features, our model can better predict hidden paths that vehicles may pass through.

### 4.1. CRF model

#### 4.1.1. Model Specification

**Figure 5:**
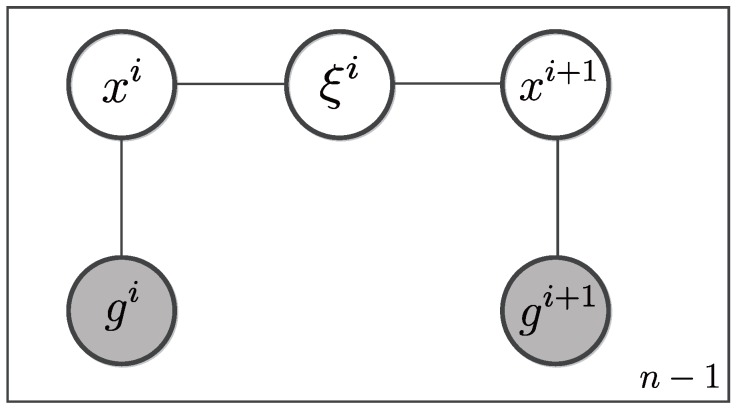
A conditional random field (CRF) model with *n* observations.

*Conditional Random Fields* (CRF) model is one of discriminative undirected probabilistic graphical models. CRF is usually used to encode relationships between observations and hidden variables. For a specific vehicle, given a sequence of GPS observations $g^{1:n}=g^{1},\cdots ,g^{n}$ and an associated path $\tau =x^{1}\xi^{1}\cdots x^{n}$, a CRF encapsulates both GPS observations and unobserved states and paths, as shown in Figure 5.

There are two kinds of cliques in this model. One includes $g^{i}$ and $x^{i}$, which represent observations and latent states respectively. The other contains $x^{i}$, $x^{i+1}$ and $\xi^{i}$, which model the relationship among latent states and paths. Instead of modeling the joint probability distribution, the CRF model intends to infer the most likely path among all candidates in the path space T, given the observations $g^{1:n}$. The conditional probability of a candidate path *τ* can be modeled as the product of factors or potential functions over all cliques. The un-normalized conditional distribution is defined as follows

(1) $$\phi \left(\tau |g^{1:n}\right)=\left[\prod_{i=1}^{n-1}\omega \left(g^{i}|x^{i}\right)\eta \left(\xi^{i},x^{i},x^{i+1}\right)\right]\cdot \omega \left(g^{n}|x^{n}\right)$$

where ω(·) and η(·) are potential functions over cliques. ω(·) describes a distribution generating noisy observation $g^{i}$ from state $x^{i}$, while η(·) defines an un-normalized joint distribution that assigns weight to the clique from state $x^{i}$ to state $x^{i+1}$ via path $\xi^{i}$.

Then we define the score of a candidate path *τ* as the normalized conditional probability as follows

(2) $$\pi \left(\tau |g^{1:n}\right)=\frac{1}{Z}\phi \left(\tau |g^{1:n}\right)$$

where *Z* is the partition function denoted by

(3) $$Z=\sum_{\tau \in T}\phi (\tau |g^{1:n})$$

Since *Z* is a constant for each candidate path *τ*, we need not calculate it in model inference. Based on this CRF model, the most likely path $\tau^{*}$ is the one with the largest conditional probability

(4) $$\begin{array}{cc} \tau^{*} & =\underset{\tau \in T}{\mathrm{argmax}}\pi (\tau |g^{1:n})\\ & =\underset{\tau \in T}{\mathrm{argmax}}\phi (\tau |g^{1:n}) \end{array}$$

#### 4.1.2. Model Inference

Two potential functions are used in Equation (1). First, function ω(·) is the observation model, representing the probability distribution of observation $g^{i}$ given the state $x^{i}$. We assume that this probability is related to the distance between $x^{i}$ and $g^{i}$, and with the *Gaussian* noise of GPS device:

(5) $$\omega \left(g^{i}|x^{i}\right)=\frac{1}{\sqrt{2\pi }\sigma }\exp\left(-\frac{|g^{i}-x^{i}{|}_{2}}{2\sigma^{2}}\right)$$

where parameter *σ* denotes the variance, and |·| denotes the Euclidean distance.

Another type of potential functions in π(·) is driver model η(·). It models the joint distribution of two adjacent states and their associated path, and assigns a weight to any possible path *ξ*. We consider the driver model to be an exponential function defined as

(6) $$\eta \left(\cdot \right)\propto \exp\left(\mu_{1}\phi_{s}\left(\xi \right)+\mu_{2}\phi_{d}\left(\xi \right)\right)$$

where $\phi_{s}$, $\phi_{d}$ are static and dynamic feature functions respectively, and $\mu_{1}$, $\mu_{2}$ are parameters.

Previous studies [8,18] usually use static feature function $\phi_{s}$ with static features such as the length of road segments, the turns of a path, the speed limit of roads, etc. There are two problems in using static features. First, it may be difficult to get those features. Second, those features are context-unaware, making the probabilistic inference not reliable especially on large sampling rate of GPS snippets. Unlike these works, we add dynamic feature function $\phi_{d}$ in Equation (6) such that both static features and dynamic context features can be utilized. Here dynamic context features denote features varying with time for different road segments, e.g., weather, road popularity, road average velocity, etc.

When the feature functions $\phi_{s}$ and $\phi_{d}$ are specified, the potential function π(·) is defined. The last issue is the inference of our CRF model, which is analogous to a dynamic programming problem that can be solved by *Viterbi* algorithm. We observe from Figure 5 that the most likely state of $x^{t}$ is determined by $\xi^{t-1}$ and $g^{t}$. If denoting the factor of those variables as $\Psi_{t}\left(x^{t},\xi^{t-1},g^{t}\right)$, the *Viterbi* recursion can be defined as

(7) $$\delta_{t}\left(j\right)=\max_{i}\Psi_{t}\left(j,i,g^{t}\right)\delta_{t-1}\left(i\right)$$

Once the latest state of *δ* is determined, we can identify the most likely sequence using the backwards recursion:

(8) $$x_{*}^{t}=\arg\max_{i}\Psi_{t}\left(x_{*}^{t+1},i,g^{t+1}\right)\delta_{t}\left(i\right)$$

After a forward and a backward recursion, the most likely path $\tau^{*}$ for GPS snippets *G* is inferred. For the further details, we refer readers to corresponding reference [8].

### 4.2. Collaborative Tensor Filter

In previous subsection we have proposed a CRF model to incorporate both static and dynamic features. In this subsection, we first introduce Tensor Filter, a collaborative dynamic feature learning model. Then we propose an effective algorithm to optimize the objective for tensor decomposition. Finally, we extract the dynamic features into the CRF path inference model.

#### 4.2.1. Tensor Construction

We firstly illustrate the key idea of collaborative feature learning model. To start with, note that most vehicles are influenced by traffic conditions spatially and temporally. We believe that a vehicle’s appearance is determined by some latent factors not only from the vehicle’s routine behavior, but also from the road traffic conditions and time stamps. Vehicles may select different paths according to the road context at different time.

Based on this intuition, we exploit a large number of GPS snippets from different vehicles to reveal latent factors among road segments, times and vehicles in a collaborative way. Unlike using matrix, we propose a tensor filter, which converts the GPS raw data into a three-order tensor A to represent the relationship among road segments, times and vehicles. Specifically, we firstly assign unique indexes to all vehicles, segments and time stamps. Secondly we fill the tensor A by values under the following rules. If a vehicle *i* appeared in road segment *j* at time stamp *k*, then (i) entry (i,j,k) is set to 1; (ii) for all segments $j^{\prime }\in E\backslash j$, entry $\left(i,j^{\prime },k\right)$ is set to 0; and (iii) otherwise if we did not observe the location of vehicle *i* at time stamp $k^{\prime }$, then entries $\left(i,j,k^{\prime }\right)$ are missing for all j∈E. Note that the original tensor A is incomplete for there are missing entries, because the GPS snippets are sparse and we could not observe the locations of all vehicles at every time stamp.

**Figure 6:**
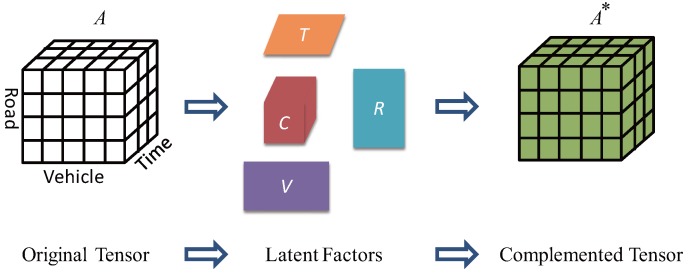
Tensor Decomposition.

The reason we assign 0 or 1 to an entry is that we consider the value of entry (i,j,k) to be the probability of vehicle *i* appeared in road *j* at time stamp *k*. For the missing entries of A, their values can be assigned a probability between 0 and 1 after tensor decomposition. As shown in Figure 6, the original tensor is sparse and a complemented tensor is derived by a tensor decomposition procedure. We can see that A is factorized into a core tensor *C* and three factor matrices: *V*,*R*, and *T*. Let the core tensor *C* multiplies factor matrices *V*,*R*, and *T* in different directions, we could get a complemented tensor $A^{*}$ where all values are filled.

#### 4.2.2. Tensor Decomposition with Regularization

To get the latent factors and complement the missing values, we conduct a tensor decomposition method as well as dealing with the data sparse problem. Assume the original three-order tensor $A\in R^{I_{1}\times I_{2}\times I_{3}}$. , where $I_{1}$, $I_{2}$ and $I_{3}$ are the number of vehicles, segments and time stamps respectively. We assign random initial values between 0 and 1 to all the missing entries of A and latent matrices. Then A can be factorized by minimizing the objective function below

(9) $$L\left(C,V,R,T\right)=\frac{1}{2}∥A-C\times_{V}V\times_{R}R\times_{T}T{∥}^{2}+R_{1}+R_{2}$$

where $C\in R^{d_{V}\times d_{R}\times d_{T}}$ is the core tensor reflecting the link between vehicles, segments and time stamps. $V\in R^{I_{1}\times d_{V}},R\in R^{I_{2}\times d_{R}},T\in R^{I_{3}\times d_{T}}$ are three latent factor matrices representing the low dimensional structure of vehicles, segments and time stamps respectively. ∥·∥ denotes the $L_{2}$ norm. The symbol $\times_{R}$ is introduced to tensor-matrix multiplication, and the subscript *R* indicates the direction of multiplication. $R_{1}$ and $R_{2}$ are regularizations.

To deal with data sparseness, reasonable regularizations need to be considered for Equation (9). We introduce the widely used $L_{2}$ norm on all latent factor matrices, which encourages the entries of of factor matrices decay to zero unless supported by data:

(10) $$R_{1}\left(C,V,R,T\right)=\frac{1}{2}\left(∥C{∥}^{2}+∥V{∥}^{2}+∥R{∥}^{2}+∥T{∥}^{2}\right)$$

In addition, we notice the observation that adjacent road segments always experience similar traffic conditions. Under this observation, a vehicle which appeared in road segment *r* at time *t*, would be likely to appear in the neighbors of *r* in recent time. Thus we normalize the topology of road network as a regularization term denoted by

(11) $$R_{2}\left(C,V,R,T\right)=\frac{1}{2}\sum_{r_{i}∼r_{j}}∥R_{i}-R_{j}{∥}^{2}$$

where $r_{i}∼r_{j}$ means road segments $r_{i}$ and $r_{j}$ are connected directly.

As Equation (9) is non-convex and there is no closed-form solution, we adopt a gradient descent method to compute a local optimum. The details are shown in Algorithm 1.

**Algorithm 1:** Tensor Decomposition Procedure **Input**: tensor A and an error threshold *ϵ* **Output**: core tensor *C*, latent factor matrices *V*,*R*,*T* 
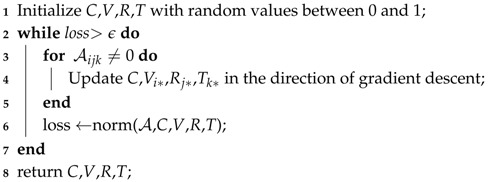


In Algorithm 1, line 4 updates the initial values to new ones that toward the direction of gradient descent. We can easily compute the derivatives of F with respect to *C*,*V*,*R* and *T*. Note that from line 4, we use an element-wise estimation instead of a batch gradient descent for efficiency [20]. The norm function in line 6 calculates the $L_{2}$ norm, denoted by the loss, of original tensor A and the new updated tensor. The procedure stops unless the loss exceeds the threshold *ϵ*. Moreover, in every iteration of the procedure, tensor-matrix and tensor-vector multiplications are needed for computing intermediate tensors and we use an open tensor toolbox [34] to get the results.

#### 4.2.3. Dynamic Feature Extraction

After tensor decomposition, latent factor matrices are derived. We can not directly use these matrices to infer path as it could not reflect vehicle’s preference. Instead we utilize the complemented tensor $A^{*}=C\times_{V}V\times_{R}R\times_{T}T$, where entry (i,j,k) indicates the probability that vehicle *i* pass through segment *j* at time stamp *k*.

For vehicle *i*, let a candidate path $\tau =x^{1}\xi^{1}\cdots x^{n}$. We denote the *k*th candidate segments set by $\xi^{k}=r_{1}^{k}|r_{2}^{k}\left|\cdots \right|r_{m}^{k}$ where $r_{i}^{k}$ is the *i*th segment composing $\xi^{k}$. We define the dynamic feature function of $\xi^{k}$ as

(12) $$\phi_{d}\left(\xi^{k}\right)=\frac{1}{N}\prod_{r_{j}^{k}\in \xi^{k}}A_{ijk}^{*}$$

where *N* is the normalization term. In fact, $\phi_{d}\left(\xi^{k}\right)$ in Equation (12) can be expressed by the probability of vehicle *i* pass through path $\xi^{k}$ at time stamp *k*. Take Equation (12) into Equation (6), we derive all potential functions for our collaborative path inference model. After model inference introduced in Section 4.1.2 for each GPS snippet, we have completed the full paths for vehicles.

## 5. Road Traffic Anomaly Detection

In this section, we aim to detect road traffic anomalies by three processes. The first process is to reconstruct trajectories from full path of vehicles (Section 5.1). Then we calculate the volume sets of vehicles to perform a Likelihood Ratio Test in Section 5.2. We give an algorithm for road anomaly detection in the end of this section.

### 5.1. Trajectory Reconstruction

To calculate the volumes of staying vehicles on each segment in recent time interval [1,T], we need to specify the locations of vehicles at every time stamp. However, the full path of a vehicle only consists of several paths and states, which could not reflect the locations at any given time stamp. Hence in this subsection we estimate the locations at any time stamp to construct fine-grained trajectories.

Assume we are given the full path $\tau =x^{1}\xi^{1}\cdots \xi^{T-1}x^{T}$, for a set of time stamps $1\leq t_{1}<t_{2}<\cdots <t_{m}\leq T$, our aim is to construct a trajectory $L=\{l_{1},l_{2},\cdots ,l_{m}\}$, where $l_{j}=\left(t_{j},r_{j},o_{j}\right)$, j∈[1,m]. To simplify the problem, we assume vehicles travel in a constant speed along sub-path between two consecutive states. Then the average velocity between states $x^{i}$ and $x^{i+1}$ along sub-path $\xi^{i}$ can be calculated by ${\bar{v}}_{i}=\frac{|\xi^{i}|}{t^{i+1}-t^{i}}$, where |·| denotes the length of path. Note that the superscript *i* indicates the time stamp of states. Let time stamp $t_{j}\in \left[t^{i},t^{i+1}\right]$, we can conclude that segment $r_{j}$ must be in sub-path $\xi^{i}$. More importantly, since we know the average velocity and the length of every segments in $\xi^{i}$, we can easily infer which segment $r_{j}$ is and the corresponding offset $o_{j}$. Then the fine-grained trajectory in time interval [1,T] is reconstructed by doing this operation iteratively. We perform the same computation on all paths of vehicles and finally derive a large number of fine-grained trajectories.

### 5.2. Likelihood Ratio Test

Given the fine-grained trajectories, we can get the volume sets of each segment in road network N. For segment e∈E, we count the volumes of staying vehicles at time stamps $1\leq t_{1}<t_{2}<\cdots <t_{m}\leq T$. We denote the volumes set as $S^{e}=\left\{V_{1}^{e},V_{2}^{e},\cdots ,V_{m}^{e}\right\}$. Then the aim of this subsection is to evaluate whether segment *e* is a SEA or CEA in this time interval.

#### 5.2.1. Self-Evolving Anomaly Detection

We utilize a *Likelihood Ratio Test* (LRT) on volumes set $S^{k}$ to detect a SEA. The LRT statistic is one of hypothesis tests that exhibit the comparison of the fitness of two models: the null model versus the alternative model. The comparison result is based on the likelihood ratio, which can be used to extrapolate how many times more likely the data are under one model than the other. The two models have identical likelihood functions, which are specified by a certain distribution with different parameters.

In this paper, the null model is associated with the hypothesis that there is no anomaly on the tested road segment, while the alternative model is associated with the hypothesis that there is an anomaly. For the volumes set $S^{e}$, we assume the items in it are independently identically distributed (i.i.d.) and follow a *Gaussian* distribution N(·|θ) with parameter θ=(μ,Σ), where *μ* and Σ are the mean volume and corresponding variance respectively. Let $\theta_{0}=\left(\mu_{0},\Sigma_{0}\right)$ be the parameters of null model which is already known. The test statistic of two models is:

(13) $$\lambda \left(e\right)=\frac{\prod_{j=1}^{m}N\left(V_{j}^{e}\;|\;\theta_{0}\right)}{\underset{\theta \in \Theta }{\sup}\prod_{j=1}^{m}N\left(V_{j}^{e}\;|\;\theta \right)}$$

where *θ* is the new parameter in space Θ that fits the observed volume set data best, and sup denotes the supremum function maximizing the likelihood distribution.

According to Equation (13), we need to compute a maximum likelihood estimate (MLE) over space Θ. To directly determine whether the alternative model is anomalous, the test statistic λ(e) is often transformed by negative twice the difference in the log-likelihoods as follows:

(14) Λ(e)=−2logλ(e)

**Figure 7:**
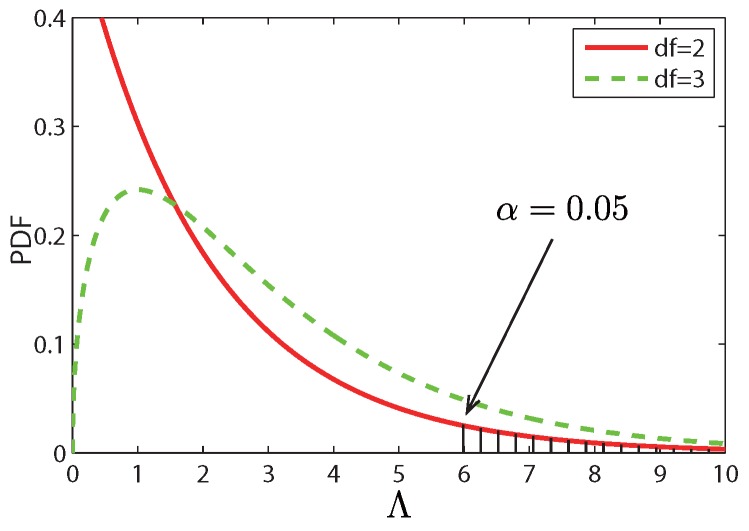
Chi-Squared Distribution.

We subsequently refer to Λ as the LRT statistic which can be approximated by an asymptotic Chi-Squared distribution $\chi^{2}\left(\Lambda ,df\right)$, where df=p−q is called the degree of freedom. p,q are the number of free parameters of the null model and the alternative model, respectively. This indicates that the Λ value of an anomaly possibly lies in the tail of $\chi^{2}$ distribution. Figure 7 shows the Probability Distribution Function (PDF) of two Chi-Squared distribution with *df* equals 1 and 3. The shaded area is the tail in which an anomaly may drop in. We define the anomaly degree of SEA as:

(15) $$d=\chi^{2}\_cdf\left(\Lambda ,df\right)$$

where $\chi^{2}\_cdf\left(\cdot \right)$ denotes the cumulative density function of the Chi-Squared distribution. Thus given a significance level *α*, if d>1−α, then the segment *e* is considered as a SEA.

Let us take an example. Suppose there is a volume set $S^{e}=\left\{85,88,94,92,96,95\right\}$. The null model is a Gaussian with parameters $\theta_{0}=\left(65,20\right)$. Then the anomaly degree of segment *e* is calculated as follows:

(a) The likelihood of null model: $L_{null}=\prod_{j=1}^{m}N\left(V_{j}^{e}\;|\;\theta_{0}\right)=2.7\times 10^{-13};$
(b) Estimate the parameter $\theta_{MLE}$ for the alternative model: $\mu_{MLE}=\frac{1}{m}\sum_{j=1}^{m}V_{j}^{e}=91.7,\;\Sigma_{MLE}=\frac{1}{m}\sum_{j=1}^{m}{\left(V_{j}^{e}-\mu_{MLE}\right)}^{2}=18.7;$
(c) The likelihood of alternative model: $L_{alter}=\prod_{j=1}^{m}N\left(V_{j}^{e}\;|\;\theta_{MLE}\right)=8.3\times 10^{-11};$
(d) Calculate Λ(e) and *d*: $\Lambda \left(e\right)=-2\log\left(\frac{2.7\times 10^{-13}}{8.3\times 10^{-11}}\right)=11.46,\;d=\chi^{2}\_cdf\left(11.46,df=2\right)=0.9968.$

Note that given a significance level α=0.05, d=0.9968>0.95. We can confidently say that segment *e* is a SEA.

#### 5.2.2. Context-Evolving Anomaly Detection

A Context-Evolving Anomaly (CEA) is a segment who significantly deviates the volumes distribution of its neighbor segments in a given region. To contrast the volume datasets, we also utilize the LRT technique. Unlike the Self-Evolving Anomaly detection, both the null model and the alternative model are not known beforehand and need to be estimated from data.

Assume the test segment *e* has a volume set $S^{e}=\left\{V_{1}^{e},V_{2}^{e},\cdots ,V_{m}^{e}\right\}$. The neighboring segments set of *e* is denoted by NS(e). For each $e^{\prime }\in NS\left(e\right)$, the corresponding volume set is $S^{e^{\prime }}=\left\{V_{1}^{e^{\prime }},V_{2}^{e^{\prime }},\cdots ,V_{m}^{e^{\prime }}\right\}$. Then the test statistic of two models is

(16) $$\lambda \left(e\right)=\frac{\underset{\theta_{0}\in \Theta }{\sup}\prod_{j=1}^{m}N\left(V_{j}^{e}\;|\;\theta_{0}\right)}{\prod_{j=1}^{m}N\left(V_{j}^{e}\;|\;\theta^{*}\right)}$$

where $\theta_{0}$ is the parameter in space Θ that best fits the observed volume set data $S^{e}$ of tested segment *e*; $\theta^{*}$ is the parameter in space $\Theta^{\prime }=\Theta -\theta_{0}$ that best fits all the neighborhood segments volume sets data of *e*. $\theta^{*}$ can be derived by maximizing the likelihood as follows:

(17) $$\theta^{*}=\underset{\theta \in \Theta^{\prime }}{\sup}\prod_{e^{\prime }\in NS\left(e\right)}\prod_{j=1}^{m}N\left(V_{j}^{e^{\prime }}\;|\;\theta \right)$$

The next phase of CEA test is similar to SEA, including the calculation of the test statistic Λ(e) and anomaly degree *d*. They are defined in Equations (14) and (15). We summarize the process of CEA detection as follows:

(a) Estimate the *MLE* parameter $\theta_{0}$ from $S^{e}$ for the null model;
(b) Calculate the likelihood of null model given $\theta_{0}$;
(c) Estimate the *MLE* parameter $\theta^{*}$ from the neighbors volume data sets of $S^{e^{\prime }}$, $\forall e^{\prime }\in NS\left(e\right)$;
(d) Calculate the likelihood of alternative model given $\theta^{*}$;
(e) Calculate test statistic Λ(e), anomaly degree *d*, and give judgement.

In the last part of this section, we summarize all the details of anomaly detection component in an algorithm called *RN-Scan*. The details are listed in Algorithm 2 as follows. Note that the functions SEA(·) and CEA(·) denote the process of examine a segment *e* by corresponding LRT defined in Section 5.2.1 and Section 5.2.2 respectively.

**Algorithm 2:** RN-Scan (Road Traffic Anomaly Detection) **Input**: Inferred vehicle path set, Road Network N **Output**: SEA set *A*, CEA set *C* 
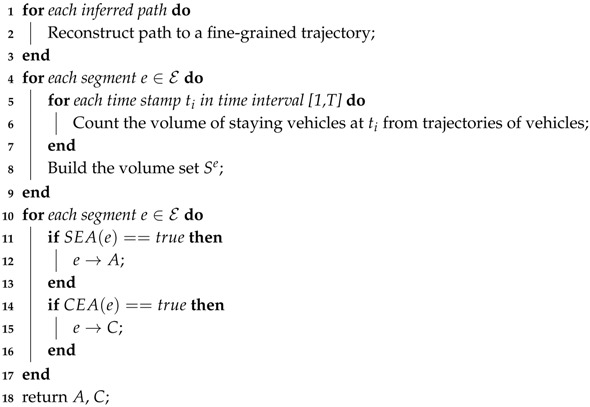


## 6. Experiments

Our experiments consist of three parts: (1) experimental settings including the data set and experimental environment; (2) the results and discussions of collaborative path inference; and (3) the evaluation effects of proposed road traffic anomaly detection algorithm.

### 6.1. Dataset

**Figure 8:**
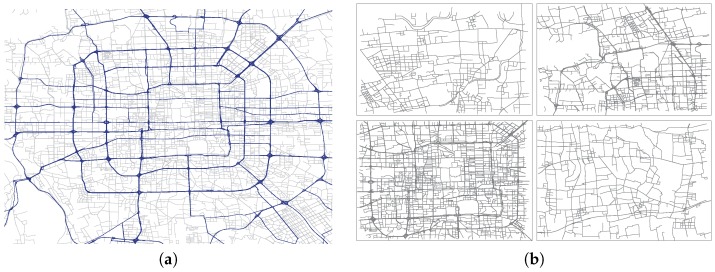
Map and Data Visualization. (**a**) Beijing Urban Area; (**b**) Selected Regions.

We use a benchmark GPS dataset, the Beijing Taxi Cab dataset which collected GPS data from 8602 taxi cabs in Beijing, China, during one month period in May 2012. It contains over 29,893,141 GPS measurements and the expected sampling rate of GPS snippets is 30 s. However, for many reasons a large number of vehicles have non-uniform sampling intervals ranging from 30 s to 10 min. Besides the GPS data, the map data used in our experiments are obtained from the Open Street Map (http://www.openstreetmap.org) of Beijing urban area, covering the whole Beijing area and storing in geo-database format. We use ArcGIS 10 as the GIS (Geographic Information System) platform to map and visualise those geographical data, as can be seen in Figure 8a.

### 6.2. Evaluation on Path Inference

#### 6.2.1. Evaluation Approach

We select four different regions in Beijing as our test regions, and each region is about 20×10 km^2^. Figure 8b displays the four regions. These regions have different functions, for example, region 3 (bottom-left of Figure 8b) is Beijing’s administrative center which is inside the Third Ring road.

For each test region, we firstly clean the map data by removing and merging some neighboring road segments to construct an undirected road network for each selected region. Then we pick out vehicles which have GPS measurements in those regions to build a GPS snippets database. We divide the GPS snippets into two subsets: a training set that all snippets are sampled within 1 min, and another test set for evaluation. Note that the test set has non-uniform intervals. To get the dynamic features, in our model both training snippets and test snippets are used to construct the tensor. We will test the effects by adjusting the proportion of training snippets versus test snippets in Section 6.2.2.

We evaluate the performance of proposed path inference method by three typical metrics: *error*, *precision* and *recall*. The error is defined as the route mismatched fraction, which refers to the ratio of the number of different segments both in inferred path *τ* and in the true path *P*, against the the total number of the true path in *P*. The precision of a path *τ* refers to the ratio of the number of true segments in *τ* against the total number of all road segments in *τ*. While the recall refers to the ratio of the number of true road segments in *τ* against the total number of true road segments *P*.

We use static CRF (SCRF) model [8] and HMM [18] model for path inference as baseline algorithms, both adopting the static features such as length of road segment. For our tensor filter, we set $\lambda_{1}$ and $\lambda_{2}$ to 0.01 for Equation (9). Next we fix the value of dimensions of latent matrices as $d_{V}=d_{T}=d_{R}=10$ . For Equation (12) we set *N* to be the number of road segments a trajectory have. In the end, we adjust parameters $\mu_{1}$ and $\mu_{2}$ in Equation (3) to get the best performance.

**Figure 9:**
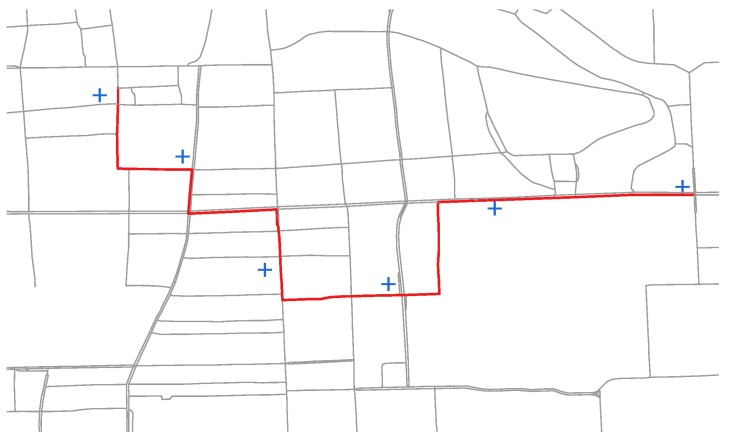
Path Labeling.

To get the ground truths of path inference, we developed a path labeling system helping experts to label the ground truth path according to incomplete GPS snippets. As shown in Figure 9, the blue cross points are GPS measurements and red line is manually labeled path. We recruited 10 experienced drivers who had 3+ years of driving experience in Beijing to label those selected snippets. Each labeler was responsible for 100 snippets and each snippet was labeled by at least 3 labelers. Then the ground truth was derived by majority voting.

#### 6.2.2. Results

**Figure 10:**
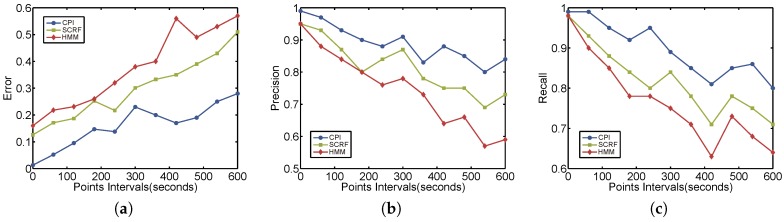
Experimental results by different sampling time intervals. (**a**) Error; (**b**) Precision; (**c**) Recall.

**Effect of time interval.** As the ratio of snippets in training set can affect the result of experiments, we first fix the ratio to 90% by selecting 450 snippets in training set and 50 snippets in test set. Then we vary the intervals of test snippets and use 5 round cross validations to get the average values. Generally the intervals of test snippets are 30 s, but we let part of GPS measurements missing to meet the test granularity requirement. Figure 10 shows the results that as the intervals between two observations grows, the performances of all methods reduce. But our collaborative method CPI (blue line) outperforms two other methods by all metrics. In detail, we can see that when intervals are lower than 2 min, the error of our CPI method is within 90% while other methods can only achieve 80%. Both precision and recall are more than 95% for our CPI method, outperforming SCRF and HMM. But the difference is not high for these three methods. This is because that the higher sampling rate of GPS snippets, the higher entropy they have for better reconstructing the original hidden trajectories. However, when time intervals increase to more than 5 min, our CPI method greatly outperforms others because it considers the dynamic context spatially and temporally. Even the intervals are 10 min, the error is lower than 30%, and the precision and recall are higher than 80% for our method. Note that if more static features are adopted, SCRF and HMM [19] could have a better performance than depicted in Figure 10, but the improvements are very limited. Our method uses dynamic context features automatically extracted from observations, with little static features but, can achieve high accuracy.

**Figure 11:**
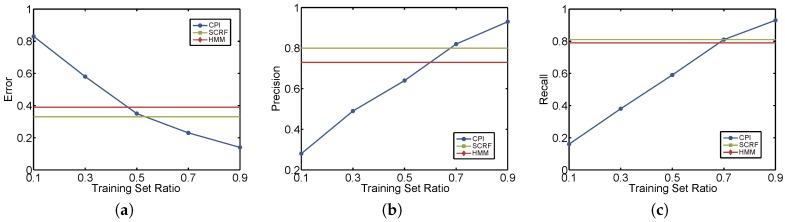
Experimental results by different training ratios. (**a**) Error; (**b**) Precision; (**c**) Recall.

**Effect of training vs. testing ratio.** We next evaluate the performance by changing the proportion of snippets of training set vs. snippets of testing set. Since the snippets in training set have small intervals, generally 30 or 60 s, the dynamic features extracted from our tensor filter would be affected seriously. But for baselines there are none of affections, as shown in Figure 11 where both the HMM and SCRF are horizonal lines. We can see that when training set ratio decreases, the error grows greatly to 80% as well as the precision and recall dropping to 0.2. When training set ratio is lower than 0.7, the precision and recall of our method are lower than the baselines. However, if the training set ratio is larger than 0.7, our method performs better. That’s because with the “help” of more high-sampling snippets in training set, the collaboration of all vehicles could do better for discovering the latent trajectories for vehicles.

### 6.3. Evaluation on Anomaly Detection

#### 6.3.1. Settings

We also use previous taxi dataset to verify the effectiveness of the proposed road anomaly detection method. Taxicabs represent one of significant components contributing the traffic volumes on the road network for big city. It is reported that about 20% of traffic on road segments in Beijing is generated by taxicabs [35]. We choose region (3) as the test region (bottom-left in Figure 8b) and retrieve all the GPS snippets of one months data for evaluation. Region (3) is within the third ring road of Beijing area. To simplify the computation, we only take the main roads into consideration and omit some low-class streets. The simplified road network of region (3) contains 185 road segments and all of them are bidirectional.

We use our collaborative path inference (CPI) model to reconstruct the incomplete GPS snippets, and count the traffic volumes for each road segment every ten minutes. Then each road segment has an associated historical volume time series.

#### 6.3.2. Evaluation for SEA

**Baseline methods.** We introduce three baseline methods: Snippet-Threshold method (ST), Snippets-LRT method (SL) and Trajectory-Threshold method (TT). Snippet-Threshold method first uses the incomplete GPS Snippets to count the volume of vehicles staying in road segments, and the *I-threshold* (an individual threshold-based method [31]) to detect road anomalies. Snippets-LRT method also uses the incomplete GPS Snippets, but the likelihood ratio test for road anomaly detection. Trajectory-Threshold method utilizes the same path inference model as proposed method to reconstruct trajectories from GPS snippets, but *I-threshold* to detect anomalies.

We evaluate the proposed method with baselines for Single-Evolving Anomaly. We select three time span for testing: (1) 8:00–9:00; (2) 13:00–14:00; and (3) 18:00–19:00. According to Equation (13), we first learn parameter $\theta_{0}$ for all segments on tested time span. Note that the values of $\theta_{0}$ for same segments are different in weekdays and weekends. In this experiment, we use the volume values in weekdays for training and testing. Since there are no ground truth anomalies in our dataset, we adopt two alternative way.

**Figure 12:**
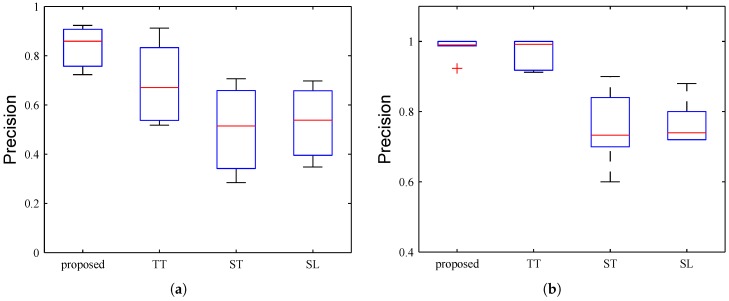
Performance of Single-Evolving Anomaly Detection. (**a**) The first way; (**b**) The second way.

The first way is volume replacement since the volumes of road segments on weekdays are significantly different from on weekends. For all segments, we take their real volumes on a weekend day to replace their corresponding real volumes on the testing weekday. Then we can safely assume all segments are SEA in the testing time span. The detection performance is shown in Figure 12a. We can see that the average precision of proposed method is nearly 90%, which is about 20% higher than the highest average precision of baseline. The variance of proposed method is also the smallest, compared to other baselines.

**Figure 13:**
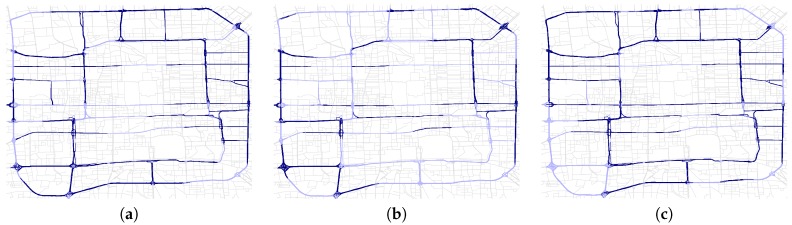
Single-Evolving Anomaly within the third ring road in different time. Segments in dark blue are anomalies. (**a**) 8:00–9:00; (**b**) 13:00–14:00; (**c**) 18:00–19:00.

Note in this scenario, the ground truths are not very accurate. The reason is that although for many time intervals the test volumes of segments are anomalous, they also exist segments whose volumes show little changes. Figure 13 presents the detected abnormal segments in different time spans. The segments in dark blue color are detected road anomalies while segments in light blue color are not. We can see that in the rush hour (Figure 13a,c) we have detected more anomalies especially on the second and third ring road, which indicates that the ring roads are heavy-traffic roads and likely to make congestions. While in the afternoon the volumes of most segments on weekdays are similar to that of weekends, which generate less road anomalies (Figure 13b).

For the second way, we use the anomaly injection technique. That is, we manually generate the volumes for tested segments, with values larger than $\theta_{0}+3\sigma$, where $\theta_{0}$ is the mean and *σ* is the standard deviation of historical traffic volumes. Thus we can treat all tested segments abnormal. The experimental results are shown in Figure 12b. The precision of proposed method achieves nearly 98%, outperforms other baselines. Although the average precision of Trajectory-Threshold (TT) method is also high, its variance is larger than our method.

#### 6.3.3. Evaluation for CEA

After the evaluation of SEA, we perform experiments for detecting Context-Evolving Anomaly (CEA). The aim of CEA detection is to find out inconsistence of road segments with their neighbor segments within a region. We tested the data in a weekday and report the number of detected anomalies in Table 1. The compared baseline is the Snippet-LRT method, which uses the original GPS snippets and the same LRT anomaly detection algorithm as ours. The results show that the proposed method can find out more Context-Evolving Anomalies than the baseline.

**Figure 14:**
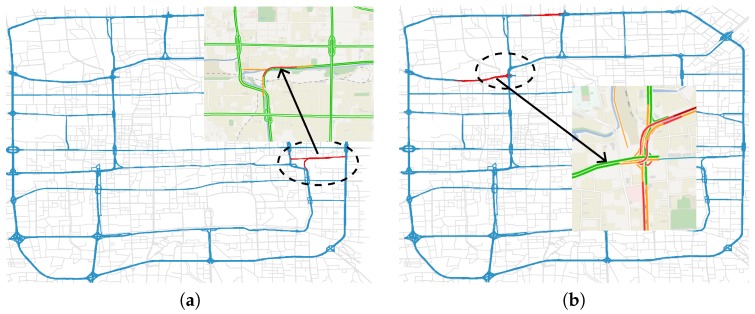
Two types of Context-Evolving Anomaly. The segments indicated by arrow is the test segments, and the area in the dotted oval is the context region. (**a**) Higher volume than neighbors; (**b**) Lower volume than neighbors.

We examine these anomalies according to the traffic reports from Beijing Traffic Management Bureau (BTMB) and find out that Context-Evolving Anomalies can be categorized into two types. For the first type, the volume of detected segment is significantly larger than its neighboring segments (Figure 14a), which means the detected segment was a traffic congestion and an event was likely to happen. For the second type, however, the volume of detected segment is significantly smaller than its neighboring segments (Figure 14b), which indicates that the detected segment may experience a road closure or it is not convenient to direct traffic flows from congested roads.

### 6.4. Efficiency

Our implemented algorithm ran on a desktop machine with Intel Core I5-3380 2.90 GHz dual core CPU and 8 GB memory. For path inference part, it would cost about 5 min to carry out the tensor decomposition. Once the dynamic context features are derived, the cost time of inference is within 2 min for all snippets in our test set. For anomaly detection part, we can detect all the road traffic anomalies within Beijing’s third ring toad in 100 ms.

## 7. Conclusions

GPS snippets collected from vehicles are characterized by noisy and sparse measurements which pose challenges on directly using them for road traffic anomaly detection. In this paper, we develop a two-stage method to effectively detect traffic anomalies from GPS snippets. To address the noisy GPS measurements, we adopt a probabilistic graphical model, i.e., Conditional Random Fields to infer the latent states and paths. The advantage of CRF model is that it is robust to noise and it can utilize both static and dynamic context features. Because of the sparse nature of snippets, dynamic features can help promote the accuracy of path inference. To get the latent dynamic features, we design a tensor factorization algorithm with reasonable regularization to collaboratively learn from snippets data. In the stage of anomaly detection, we define and recognize two types of road traffic anomalies, namely SEA and CEA, and propose an algorithm called RN-Scan to effectively find them. Our algorithm leans on the Likelihood Ratio Test (LRT) superseding traditional threshold based method to evaluate the anomaly degree for each segment in road network. In view of real-world experimental results, we discover that our method can effectively achieve the proposed objects, both on path inference and road anomaly detection. We believe that our work will not only advance the research on IoT and urban computing, but also benefit many real-world location-based applications.

However, there could be other road traffic abnormal patterns, such as normal volume of traffic with much slower velocity than usual, or several faraway segments behave abnormal on volumes. A thought is that other traffic pattern related information could be included to the tensor decomposition for refined dynamic feature discovery. Further more, we could collect multi-source location-based datasets such as taxicabs, check-ins, smart card records, etc. Those data could also be utilized to discover abnormal traffic patterns from different points of view. We could combine them into one model and perform multi-task learning to jointly detect traffic anomalies. We leave all these problems for future work.

## Figures and Tables

**Table 1:** The number of detected CEA in a day.

	8–10	11–13	14–16	17–19	20–22
proposed	7	6	4	5	5
Snippet-LRT method	3	3	4	1	3
